# Electrospun Structures Made of a Hydrolyzed Keratin-Based Biomaterial for Development of *in vitro* Tissue Models

**DOI:** 10.3389/fbioe.2019.00174

**Published:** 2019-07-17

**Authors:** Gabriele Maria Fortunato, Francesco Da Ros, Samuele Bisconti, Aurora De Acutis, Francesco Biagini, Anna Lapomarda, Chiara Magliaro, Carmelo De Maria, Francesca Montemurro, Dario Bizzotto, Paola Braghetta, Giovanni Vozzi

**Affiliations:** ^1^Research Centre ‘E. Piaggio', Department of Ingegneria dell'Informazione of University of Pisa, Pisa, Italy; ^2^Department of Molecular Medicine, University of Padova, Padova, Italy

**Keywords:** hydrolyzed keratin, gelatin, GPTMS, TFE, electrospinning, tissue engineering

## Abstract

The aim of this study is the analysis and characterization of a hydrolyzed keratin-based biomaterial and its processing using electrospinning technology to develop *in vitro* tissue models. This biomaterial, extracted from poultry feathers, was mixed with type A porcine gelatin and cross-linked with γ-glycidyloxy-propyl-trimethoxy-silane (GPTMS) to be casted initially in the form of film and characterized in terms of swelling, contact angle, mechanical properties, and surface charge density. After these chemical-physical characterizations, electrospun nanofibers structures were manufactured and their mechanical properties were evaluated. Finally, cell response was analyzed by testing the efficacy of keratin-based structures in sustaining cell vitality and proliferation over 4 days of human epithelial, rat neuronal and human primary skin fibroblast cells.

## Introduction

In order to reproduce a functional engineered *in vitro* tissue model, it is important to mimic the topological and mechanical features of native tissue, given the strict relationship between function and structure (Vozzi and Ahluwalia, 2007). Human tissues are complex three-dimensional (3D) structures composed of different cell types arranged in complex topologies where a well-organized vascular network ensures that the distance of each cell from a vascular capillary is <50 μm. Moreover, since each tissue presents a well-defined mechanical behavior, inappropriate mechanical properties will induce abnormal cell activities. *In vivo*, cells are interconnected each other in the 3D volume and not in a plane as it normally happens in the standard cell culture in a multi-well plate. On the basis of this evidence, it is important to create 3D polymeric scaffolds able to guide cell growth and activation of their functions.

Several synthetic and natural biomaterials have been tested and modified in order to mimic extra-cellular matrix (ECM) characteristics. Traditionally, synthetic polymers have been used because they can be processed more easily with different bioprinting technologies in different complex structure in order to mimic at meso- and micro- level the properties of targeted tissues (De Maria et al., 2017; Moroni et al., 2017). However, their degradation once tested *in vitro* or *in vivo* does not ensure their correct subsistence for the entire period required by cells to colonize the structure, because the degradation mechanism is often not controllable and its products can alter cell processes and sometimes can induce inflammatory process. Contrary to synthetic polymers, natural polymers (usually extracted from natural tissues) match all the properties required to mimic ECM and degrade much faster than synthetic biomaterials, even if sometimes they are difficult to process with bioprinting technologies.

Keratin is an interesting natural biomaterial that can be used for scaffold fabrication. It is a fibrous protein with a structure similar to collagen, and it is the main component of the epidermal corneal layer, nails and appendages such as hairs, horns, feathers, and wool. Keratin contains many molecules of the aminoacid cysteine that enables disulfide bridges formations. Disulfide bridges boost the hydrogen bonds action in maintaining a close cohesion of keratin chains, wrapping in a structure similar to a helix. These bonds, provide stiffness and strength to hair, nails and horns (Zhang et al., 2014).

During the extraction process, keratin is degraded, chemically or enzymatically, in units of smaller dimensions with the breaking of disulphide bridges (hydrolyzed keratin) thereby losing its characteristics of rigidity and insolubility in water. Only through enzymatic hydrolysis, it is possible to control and obtain subunits of desired molecular weight (Tsuda and Nomura, 2014; Staroszczyk and Sinkiewicz, 2017).

Keratin and its hydrolizates have tripeptides sequences Arg-Gly-Asp (RGD) and Leu-Asp-Val (LDV) that could bind with cell surface ligands acting as the ECM that facilitates cell-cell and cell-matrix interactions promoting cell adhesion and supporting cell proliferation (Wang et al., 2012; Burnett et al., 2013). Thanks to these properties, keratin and hydrolized keratin derived from by-products of animal industry, such as wool fibers, animal hair and feathers, have been processed alone or in combination with other biomaterials to form film, sponges and scaffolds for medical applications (Rouse and Van Dyke, 2010) such as wound healing, bone regeneration (Saravanan et al., 2013), drug delivery (Saul et al., 2011), haemostasis, and peripheral nerves repair (Wang et al., 2012).

The weak point of keratin-based materials is their poor mechanical properties (Yamauchi et al., 1996), resulting too fragile for practical use. This issue can be solved by the addition of natural or synthetic polymers such as chitosan, silk fibroin, gelatin, and polyethylene oxide (Prasong and Wasan, 2011; Balaji et al., 2012). Chitosan, in particular, is derived from chitin, that is the second most abundant natural polymer. Chitin-based materials are widely used in the biomedical field thanks to their properties of biocompatibility and biodegradability, but also to their cationic nature that enhances the interaction with growth factors, nucleic acids and cytokines. Main tissue engineering applications of chitin are related to the regeneration of bone, while they were not completely examined for skin and soft tissues (Yang, 2011).

Homogeneous films with smooth surfaces were obtained, from mixtures of keratin and gelatin, which showed a high miscibility between the two components (Rouse and Van Dyke, 2010; Prasong and Wasan, 2011). If provided with the right porosity (Zeltinger et al., 2001; O'Brien et al., 2005), scaffolds made with gelatin and keratin could be able to mimic ECM microenvironment, characterized by high pore interconnectivity and a pore size which ranges from meso to nano scale. In recent years, electrospinning has emerged as an enabling tool for fabricating scaffold with these multiscale features (Soliman et al., 2010; Sreerekha et al., 2013).

In this work, we produced and characterized films made of hydrolyzed-keratin-based biomaterials in terms of their chemical and physical features (swelling, contact angle, mechanical properties, and surface charge density). These materials were processed using the electrospinning technique to develop a suitable substrates for *in vitro* tissue models, which were tested with different cell types. This method allows to produce a thin polymeric structure that presents an intrinsic micro and nano porosity similar to the one of the natural ECM. Dimensions of the fibers, porosity and topology of the structure can be modulated varying working parameters, e.g., the distance between the metal needle and the collector or the applied voltage, and the solution parameters, e.g., the polymer concentration (Subbiah et al., 2005; Carrabba et al., 2016).

## Materials and Methods

### Keratin Based Solutions and Film Preparation

Hydrolyzed keratin was gently provided by Consortium SGS (Santacroce sull'Arno, Italy), a company that processes animal byproducts. In this specific case, keratin was obtained from chicken feathers processed by alkaline hydrolysis (MW > 10 kDa) containing about 1% of free amino acids. The final concentration was 35° Brix.

A 10% w/v aqueous solution of gelatin from porcine skin, type A (Sigma-Aldrich, Italy) was prepared dissolving gelatin powder by continuous stirring at 50°C for 1 h. (3-Glycidyloxypropyl)-trimethoxysilane (GPTMS) (Sigma-Aldrich, Italy) was used as cross-linking agent.

Gelatin (G) and keratin hydrolizates (K) solutions were mixed at different volume ratios: K:G 1:1, 1:2, 2:1. GPTMS was added in a ratio of 6% w/v respect to the final volume, and the resulting solutions were stirred for 40 min at 50°C. Films from the three different solutions were prepared by casting in a plastic petri dish of 3 cm diameter and dried for 24 h at 37°C.

### Electrospinning Material Preparation

Solutions for electrospinning were prepared starting from a 10% w/v gelatin type A solution in 50% w/v (in deionized water) 2,2,2-Trifluoroethanol (TFE) (Sigma-Aldrich, Italy) (Ki et al., 2005; Zhang et al., 2005; Choktaweesap et al., 2007; Mindru et al., 2007; Song et al., 2008; Chen et al., 2009; Elliott et al., 2009; Nguyen, 2010; Soliman et al., 2010; Zhan and Lan, 2012; Tamrin Nuge, 2013; Dai et al., 2014) by stirring at room temperature for 24 h. TFE was chosen due to long term stability of keratin hydrolizated solution, which tends to form a precipitate, impossible to be electrospun with other solvents. Keratin hydrolizates and 6% w/v GPTMS were added to this gelatin solution according to three different volume ratios used for film preparation (K:G 1:1, 1:2, 2:1).

### Swelling Test on Films

Three samples for each type of film were dipped in deionized water and at specific time points (every 30 min for the first 4 h, then every 1 h until 6 h and finally at 24 h, 30 h, and 4 days) their weight (using a Metler Toledo AE240 balance) and area (using ImageJ software after image acquisition with Canon A620 digital camera) were measured. The swelling percentage was calculated according to Equation (1):

(1) $$\%swelling=100\times \frac{p_{i}-p_{0}}{p_{0}}$$

where p_i_ is the weight (or area) measured at the *i-th* time point and p_0_ is the initial weight (or area). Then, samples were dried and weight loss percentage was measured (Equation 2):

(2) $$\%weight\;loss=100\times \frac{p_{0}-p_{f}}{p_{0}}$$

where p_f_ is the final weight of dried samples.

### Contact Angle Measurement on Films

Static contact angle was measured using the sessile drop method, with a 5 μl double distilled water droplet at room temperature. Images were acquired with a horizontal optical microscope equipped with a digital camera and the CAM 200 software. The test was performed on dried and hydrated samples; for each angle reported, at least five measurements on different surface locations were averaged.

### Surface Charge Density Measurement

**Figure 1:**
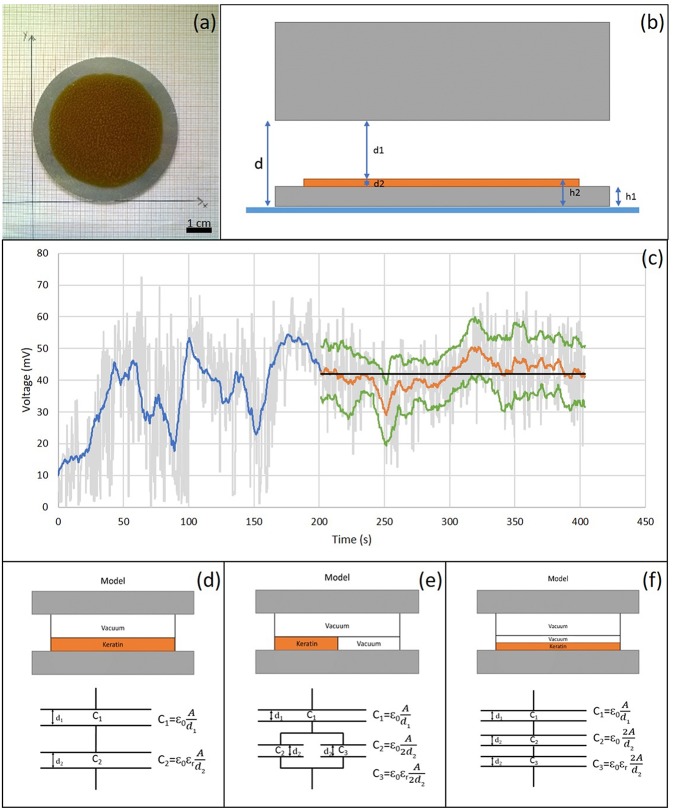
**(a)** Casted K:G 1:1 solution on a metal plate. Scale bar = 1 cm; **(b)** Schematic structure of the measurement system; **(c)** Example of data analysis. In gray voltage trend detected by Matlab developed software, in blue the application of the moving mean filter, in orange the section with mostly constant trend considered and, in green the relative standard deviation. The constant stretch in black represents the average value; **(d)** Standard model: uniform K:G solution; **(e)** Model 1: parallel model between K:G solution and air bubbles, modeled with the same properties of vacuum; **(f)** Model 2: series model between K:G solution and air bubbles.

Surface potential or charge can have influence on cell adhesion (Chang et al., 2014, 2016). Surface potential measurements were made using a Kelvin vibrating plate (KSV Instruments, Sweden), with a nominal error of +/- 10 mV. The Kelvin vibrating plate measures the surface potential between two conductors, the sample holder and probe respectively, placed about 1 mm apart. The difference in measured potential between the sample holder with and without (reference potential) the sample is given by the surface potential of the sample, and it depends on its thickness, dielectric constant and surface charge density. For these experiments the keratin-based solutions were casted on aluminum plates (Figure 1a). Three samples of each solution were analyzed. After the acquisition of the reference potential (i.e., sample holder without sample), the thickness of casted film and the distance between the film and the vibrating plate (according to the diagram shown in Figure 1b) were measured. The potential difference was evaluated every 10 min using a purposely written software, developed in Matlab®. Data were filtered with a moving average filter and the variance analysis was performed in order to determine when the signal presented a constant trend (Figure 1c), which was taken to evaluate the surface charge density of polymer.

The surface charge density was calculated by modeling the system as a capacitor, with three different configuration reported in Figures 1d–f. In the first model (Figure 1d), the material was considered to be homogeneous while, in the cases shown in Figures 1e,f, the contribution of the air bubbles, that can be present in the film (~50%), was taken into account.

### Electrospinning Process

**Figure 2:**
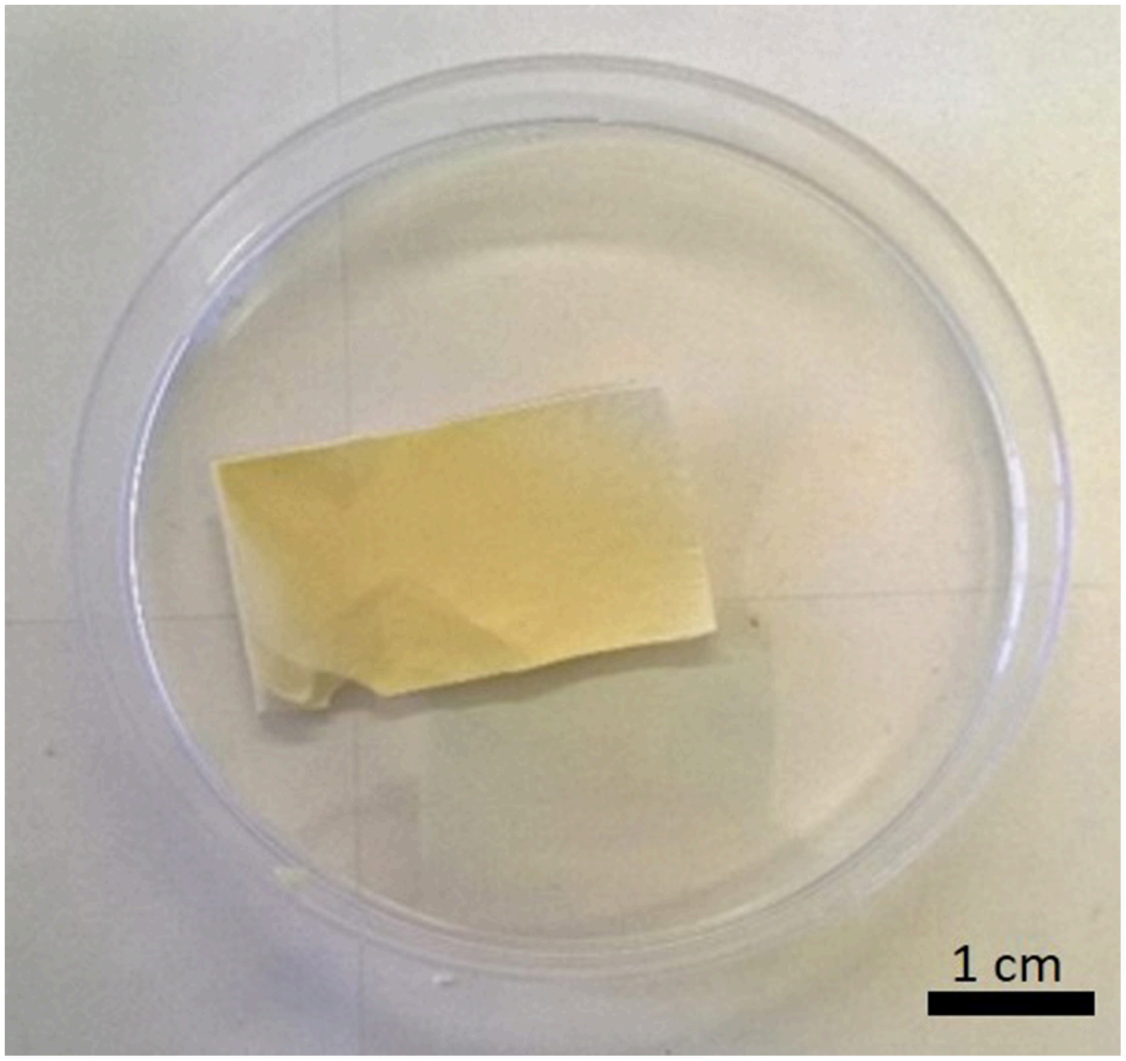
K:G 1:1 sample (2 h deposition). Scale bar = 1 cm.

In this work, electrospun structures were fabricated from the three different solutions using a Linari electrospinning system (Linari Engineering Ltd., Pisa, Italy), by varying the applied potential, the flow rate, the distance between the needle and the collector and the electrospinning process duration (time), in order to find the optimal working parameters (Table 1). For each one of the 81 combinations of the parameters, three samples were fabricated (example in Figure 2).

**Table 1:** Investigated electrospinning parameters.

**Flow rate (ml/h)**	**1**	**2**	**3**
Applied potential (kV)	20	30	40
Distance from collector (cm)	10	15	20
Time (h)	1/2	1	2

### Mechanical Characterization

Mechanical characterization of both casted films and electrospun structures was carried out performing uniaxial tensile tests using a universal machine Zwick-Roell Z005 ProLine equipped with a 100N load cell. Three rectangular shaped specimens for each type of structure were tested. Samples were pulled with a strain rate of 10%/min of the initial length until failure. Load F (N) and elongation Δx (m) were recorded and from the stress–strain graphs the elastic modulus E (Pa), the failure stress σ_max_, (Pa), the corresponding failure strain ε_r_, and toughness U (J m^−3^) were calculated.

Regarding electrospun structures, to evaluate how variations in the process parameters affect the mechanical properties, four different cases (Table 2), were considered: in each case, one parameter a time varies (K:G ratio, Flow (ml/h), Voltage (kV), electrospinning duration (Time, h), while the distance was kept constant.

**Table 2:** Combination of different electrospinning parameters analyzed to evaluate their effect on mechanical properties of keratin-based structures.

	**Test**	**K:G ratio**	**Distance (cm)**	**Flow (ml/h)**	**Voltage (kV)**	**Time (h)**
Case 1	V1	1:2	20	1	30	2
	V2	1:2	20	1	40	2
Case 2	T1	1:1	20	3	30	1/2
	T2	1:1	20	3	30	1
	T3	1:1	20	3	30	2
Case 3	F1	1:1	20	1	30	2
	F2	1:1	20	3	30	2
Case 4	C1	1:2	20	1	30	2
	C2	1:1	20	1	30	2

*For each case, the values of the parameter under investigation are underlined*.

### Scanning Electron Microscopy and Image Analysis

The microscopic structure was analyzed by the scanning electron microscopy (SEM), focusing attention on diameter and distribution of the fibers. Three images were acquired for each sample at 300×, 500×, and 1,000× magnification. A qualitative diameter analysis was carried out by randomly measuring fibers in acquired images (at 1,000× magnification) by ImageJ software.

### Cell Vitality and Proliferation Assay

Cell culture was performed on K:G 1:1 electrospun samples given their mechanical properties more similar to biological tissues (see section Results), and the following electrospinning parameters were selected: 30 kV, 20 cm, 1 ml/h, 1 h. Human epithelial HEK-293T cells, rat neuronal RT4-P6D2T cells and human primary skin fibroblasts were plated at density of 2 × 10^4^ cells/well in a 12-well plate on keratin-based structure and cultured in DMEM-10%FBS (Invitrogen) and evaluated at three time points (1, 2, and 4 days).

For proliferation assay, 5-ethynyl-2′-deoxyuridine (EdU, 10 μM in PBS, Invitrogen) was added in the medium 12 h before the end of the experiment. To monitor cell vitality, CellTracker ™Green CMFDA (5 μM in PBS, Invitrogen) was added for 1.5 h before fixation. Cells were fixed in 4% PFA for 5 min, washed twice in PBS for 5 min and then stained for EdU with Click-IT EdU Alexa Fluor 555 Imaging Kit (Life Technologies). Hoechst 33258 (Sigma Aldrich) was used as nuclear counterstain. Immunofluorescence acquisition was performed using Zeiss LSM 700 confocal microscopy.

### Statistical Analysis

A Shapiro-Wilk test was performed to evaluate if data distributions were normal. Statistical differences between Gaussian groups were evaluated by one-way ANOVA. Having three groups, in case of the existence of a significant difference, a *post-hoc* analysis using the Tukey test was carried out, to check the difference between each pair of means. For cell vitality and proliferation, statistical analysis was performed with one-tail Mann-Whitney test. For each statistical test a significance level *p* = 0.05 was considered. Correlation was evaluated by Pearson coefficient (r) considering a direct/inverse proportionality with |r| > 0.7.

## Results

### Swelling Test

**Figure 3:**
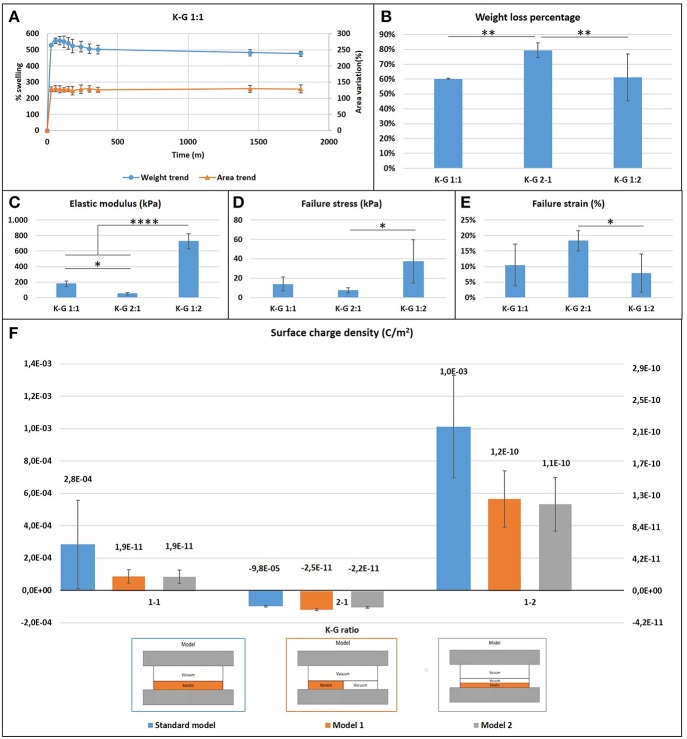
**(A)** Casted samples K:G 1:1 swelling trend for weight and area (samples K:G 1:2 and K:G 2:1 showed a similar trend); **(B)** Weight loss percentage; Tukey's multiple comparisons test ^**^p < 0.007; **(C)** Elastic modulus trend; ^*^p < 0.02, ^****^p < 0.0001; **(D)** Failure stress trend; ^*^p < 0.02; **(E)** Failure strain trend; ^*^p < 0.04; **(F)** Surface charge density for three different model.

Films presented a large initial (within the first hour) swelling, which increases with the keratin concentration presenting a weight increase of 400% in the case of K:G 1:2 and 700% in case of K:G 2:1, before reaching a plateau after 6 h (Figure 3A). This result suggest that the hydrolized keratin favors the inlet of water inside the sample. The increase of geometrical dimensions (area) follows the same trend of weight variation.

Samples with higher keratin content present a higher weight loss (around 80%) respect to samples with a lower content of keratin (60%) (Figure 3B). A one-way ANOVA showed a statistically significant difference between groups (*F* = 19.54, *p* = 0.002 < 0.05).

### Contact Angle Measurement

Both hydrated and dried samples present an high hydrophilicity, with a low contact angle (<40° for dry samples and <8° for wet samples). The influence of keratin content is significant only in hydrated sample (*F* = 7.054, *p* = 0.014 < 0.05), while no statistical differences were noted in dry samples (*F* = 3.095, *p* = 0.095).

### Surface Charge Density Measurement

The measure of the surface charge density shows negative values where the keratin content prevails, regardless the type of mathematical model used for analyzing data (Figure 3F). This behavior can be explained considering the isoelectric point of the components. The starting solution is in fact strongly basic with a pH~13 and well above the isoelectric point of keratin (~9) and gelatin (~7), and thus in solution both proteins present a negative charge The addition of GPTMS decreases however the negative charge (Kamra et al., 2015; Rosaria et al., 2017). The presence of air bubbles strongly affects the calculus of surface charge density: if the content of air is neglected the models indicate a density which ranges from 10^−3^ to 10^−5^ C/m^2^, while it results between 10^−10^ and 10^−11^ C/m^2^ if air is taken into account.

### Electrospinning Processing and Topological Analysis

The keratin and gelatin based solutions resulted suitable for electrospinning, forming 3D structures with variable thickness depending on set parameters: for example, an increase in deposition time or flow rate increases the thickness. The diameter of the fibers was instead evaluated by SEM images analysis. The examined samples were electrospun at the same flow rate and deposition time, and thus these parameters were excluded from the analysis of the results.

**Figure 4:**
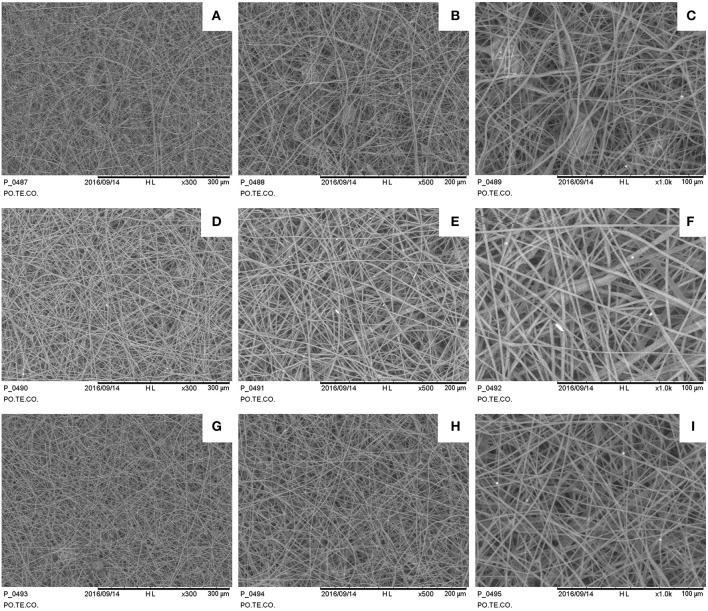
SEM images for different electrospun samples (1 h deposition at 1 ml/h): **(A–C)** 35 kV, 15 cm sample 300×, 500×, 1,000×; **(D–F)** 35 kV, 20 cm sample 300×, 500×, 1,000×; **(G–I)** 50 kV, 15 cm sample 300×, 500×, 1,000×.

Samples show a decrease in the diameter of the fibers as the applied voltage increases (Figure 4), from 1.73 ± 0.35 μm at 35 kV to 1.01 ± 0.32 μm at 50 kV (a one-way ANOVA showed a statistically significant difference between different samples: *F* = 9.232, *p* < 0.001). Some defects are present mainly consisting in pearl-shaped structures in samples fabricated at 35 kV. The needle-collector distance seems to not affect the fiber dimensions.

### Mechanical Characterization

Mechanical characterization of casted wet samples showed that (Figures 3C–E):

1. the elastic modulus and maximum stress increases as the gelatin content increases (one-way ANOVA on elastic modulus data: *F* = 166.2, *p* < 0.0001; maximum stress: *F* = 6.316, *p* = 0.017);
2. the failure strain decreases as the gelatin content increases (*F* = 4.643, *p* = 0.038).

These statistically significant differences indicate that the increase of gelatin content creates stiffer but more fragile biomaterials. Mechanical properties of electrospun films were evaluated according to different cases showed in Table 2.

**Figure 5:**
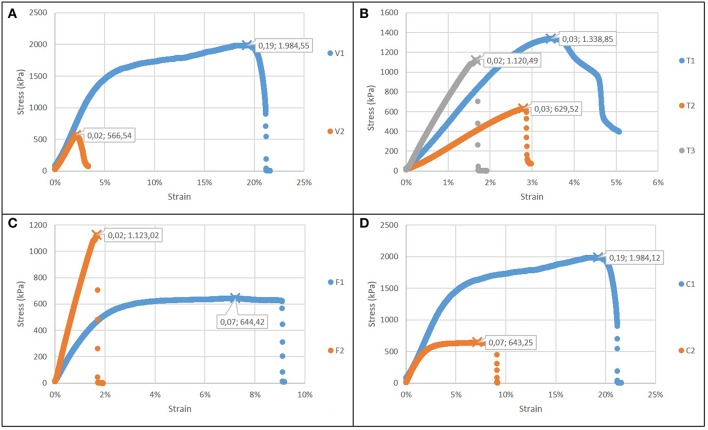
Stress strain curves for electrospun films (in each graph ε_r_ and σ_max_ are showed): **(A)** Case 1: different voltage applied; **(B)** Case 2: different time of deposition; **(C)** Case 3: different flow rate; **(D)** Case 4: different type of solution.

Figure 5A reports the comparison of case 1: the increase of the voltage forms a more brittle and less resistant material, with a reduced failure stress and strain, but not significant changes in the elastic modulus. The increase in electrospinning process duration (Figure 5B) results into stiffer but less compliant biomaterial, with a lower toughness. An increase in flow rate results also in greater stiffness and lower toughness (Figure 5C). The gelatin content also influences the properties of the structure (Figure 5D): as its content increases, the sample is more resistant (as in casted samples), but it is also less brittle, indicating an influence of the micro and nanostructure. In all cases, the elastic modulus was in the range 30–70 MPa.

### Cell Vitality and Proliferation Assay

To test the efficacy of keratin-based structure in sustaining vitality and proliferation of cells we adopted different approaches.

**Figure 6:**
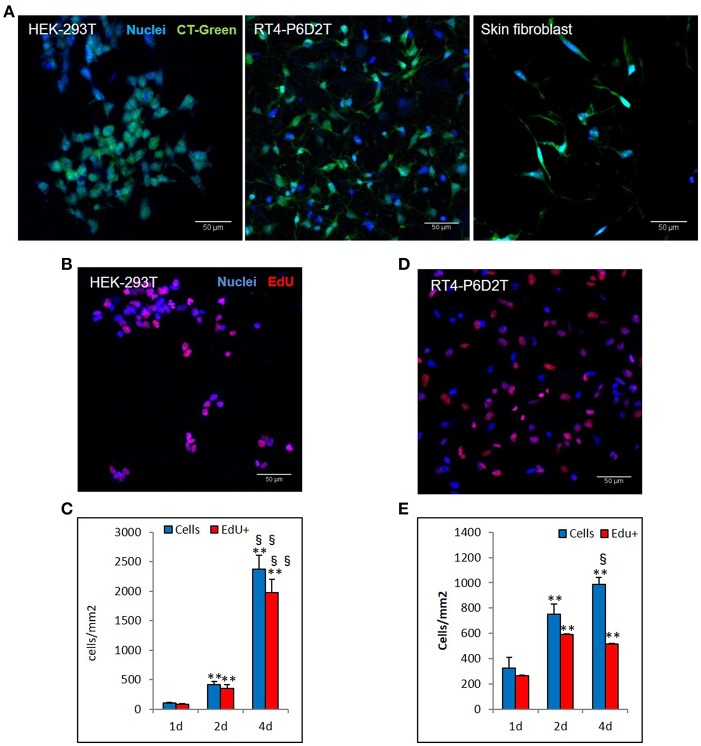
Different cell lines are vital and proliferate in keratin based structure. **(A)** CellTracker ™Green CMFDA staining (CT-Green) of human HEK-293T, rat RT4-P6D2T cells and human primary skin fibroblasts after 4 days of culture. Hoechst was used for nuclear staining. Scale bar 50 μm; **(B,D)** Representative images after 2 days of culture of EdU staining (red) of HEK-293T **(B)** and RT4-D6P2T **(D)** cells. Hoechst was used for nuclear staining. Scale bar 50 μm; **(C,E)** Histograms showing the total number of cells and EdU positive cells for square millimeter after 1, 2 and 4 days of culture. ^**^/§§*P* < 0.01; ^*^ vs. 1d, § vs. 2d.

**Figure 7:**
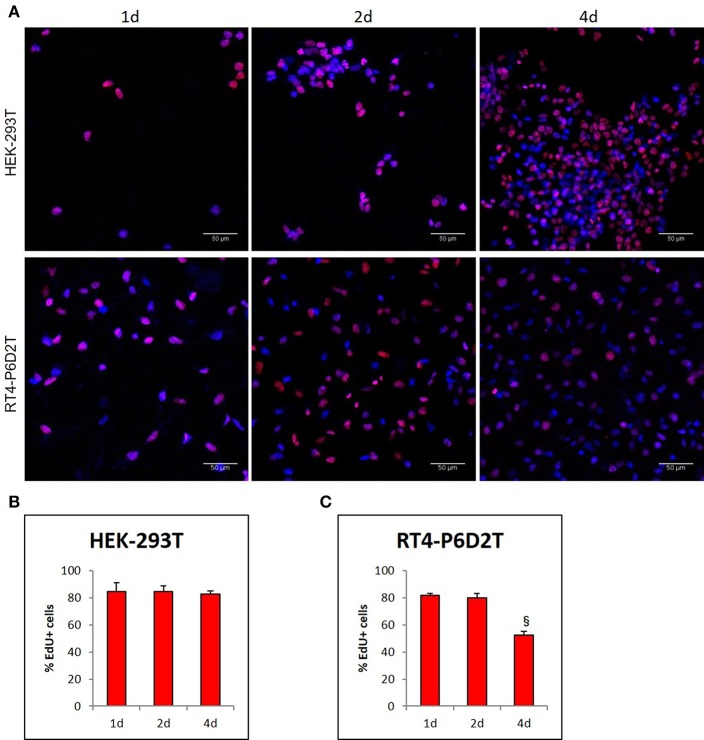
**(A)** Representative images of EdU staining in HEK-293T and RT4-D6P2T cells after 1, 2 and 4 days of culture on keratin based structure; **(B,C)** Histograms representing the percentage of HEK-293T **(B)** and RT4-P6D2T **(C)** EdU positive cells/total cells. Hoechst was used for nuclear staining. §*P* < 0.05 vs. 2d. Scale bar is 50 μm.

**Figure 8:**
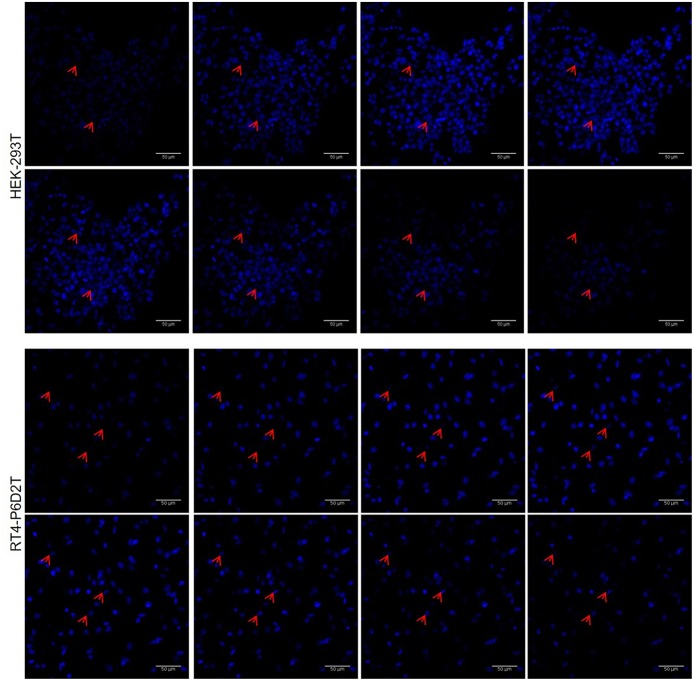
Representative image sequence for HEK-293T and RT4-P6D2T nuclei after 4 days of culture, showing cells growing on different planes. Red arrows indicate some nuclei belonging to cells grew on different planes in keratin bases structure. Hoechst was used for nuclear staining. Scale bar is 50 μm.

Firstly, we used the CellTracker Green dye to test if different types of cells are vital after 4 days of culture in the keratin based structure. This fluorescent probe was designed to freely pass through cell membranes, but it is transformed in a cell-impermeant reaction product only in vital cells. As shown in Figure 6A, all of the different cell types considered (human epithelial HEK-293T, rat neuronal RT4-D6PD2, human primary skin fibroblasts) showed a strong fluorescent staining after 4 days of culture, meaning they are truly vital. Moreover, to better understand if the biomaterial sustains also the proliferation, and not only the vitality of cells, we performed proliferation assay using EdU dye at three different time points. Both HEK-293T (Figures 6B, 7A) and RT4-D6PD2 (Figures 6A–D) showed high proliferation rate (EdU+ cells) at all the three time points (Figures 7B,C). An increase in the total number of cells per squared millimeter is evident already at day 2 and it continues to raise at 4 days (Figures 6C–E, 7A). Furthermore, cells were well-distributed in the three dimensions within the biomaterial as demonstrated by the variation in nuclei positions that could be observed in different plane in a Z projection obtained by confocal microscopy (Figure 8).

## Discussion

In this work we presented a method for the use of a waste material such as keratin extracted from poultry feathers and its potential applications in tissue engineering field.

Results from casted samples characterization indicates that we can tune the chemical and mechanical properties of keratin-based biomaterials by varying the ratio between gelatin and keratin content, keeping the GPTMS content constant. Samples with higher keratin content present a better ability to adsorb water, which can be addressed to a less dense mesh of intermolecular bonds, while increasing the gelatin content let to a more dense molecular mesh with a lower weight loss and a lower swelling. These conclusions are also confirmed by mechanical testing, which indicates more rigid samples with increased gelatin content. The surface charge density test highlighted a difference between the different types of solutions. In particular, as the keratin content increases the surface charge density becomes more negative due to the isoelectric point of the two materials as explained in the section Results.

With regard to the electrospun structures, the main results obtained from the mechanical characterization can be summarized as follows:

1. as the flow rate increases and the duration of the electrospinning process is extended, stiffer but considerably more brittle structures are obtained;
2. with a higher percentage of gelatin, there is an increase in the strength and toughness of the fiber matrix, as showed by casted films;
3. the increase in voltage leads to embrittlement and lower strength of the fibers;
4. the elastic modulus of the nanofiber matrix is directly proportional to the electrospinning time and the flow rate, while it is not influenced by the voltage and the gelatin percentage of the solution;
5. the maximum stress is inversely proportional to the voltage and directly proportional to the percentage of gelatin present in the solution;
6. the failure strain is influenced by all the parameters of the electrospinning process. In particular, it is inversely proportional to the voltage, the duration of the test and the flow rate, while it increases as the percentage of gelatin increases;
7. the energy per unit of volume stored by matrix (i.e., toughness) decreases with increasing voltage and flow rate.

Main results of mechanical characterization are summarized in Table 3.

**Table 3:** Mechanical characterization on electrospun samples results: ↑means direct proportionality, ↓means inverse proportionality, – means that there is no correlation between parameters.

**Parameters**	**Elastic modulus**	**Failure stress**	**Failure strain**	**Toughness**
Voltage		**↓**	**↓**	**↓**
% gelatin		**↑**	**↑**	**↑**
Electrospinning duration	**↑**		**↓**	**↓**
Flow	**↑**	**↑**	**↓**	**↓**

Furthermore, based on the effects that each parameter of the electrospinning process has on the mechanical properties of the nanofiber matrices, it is possible to choose which combination of parameter is the best according to different applications. For example, when regenerating biological tissues, it is preferable that the mechanical characteristics of the electrospun structures are as close as possible to those of the tissue to reproduce. In particular, the results obtained from the mechanical characterization are comparable to two specific soft tissues, such as nerve tissue and skin. Comparing skin values (Dunn and Silver, 1983; Khatyr et al., 2004; Jacquemoud et al., 2007; Ní et al., 2012) with those obtained from tensile test of the matrices of nanofibers, it was noticed that the elastic modulus of the electrospun structures (30–70 MPa) were comparable with skin values, even if with lower ability to resist to large deformation.

Considering, instead, the nervous tissue and its mechanical properties (Kamra et al., 2015), results showed that electrospun structures K:G 1:1 are stiffer than the biological target, even if failure stress and strain are similar. Reducing the deposition time or flow rate can be an option to mimic in this case the biological tissue. These mechanical properties affect cell response and vitality. It was demonstrated that electrospun constructs are biocompatible and can be colonized by human epithelial cells, rat neurons and primary fibroblasts of human skin due to their high porosity. However, due to too different stiffness values compared to nervous tissue, epithelial cells resulted more proliferating after 4 days compared to neural cells, thus showing promising results for applications for the regeneration of epithelial tissue. Many other biomaterials have been tested in recent studies for skin and nerve regeneration. For example, natural polymers such as collagen, gelatin, chitosan and hyaluronic acid have been widely investigated; however, these materials present some disadvantages such as low stability in aqueous environment, difficulty to be electrospun or poor mechanical properties. To overcome these limitations, these polymers are often used in combination with synthetic materials such as Poly(lactide–*co*–glycolide) (PLGA), Poly(ε-caprolactone) (PCL), Polyurethane (PU), or Poly(L–lactide) (PLLA); however, using these polymers can reduce scaffolds cytocompatibility and biodegradability (Ladd et al., 2012; Liu et al., 2013; Sundaramurthi et al., 2014). In our work, instead, two natural polymers such as keratin and gelatin were used, thus enhancing these two aspects previously cited. In the future, it will be necessary to investigate how cell culture and extra cellular matrix production may affect the mechanical characteristics of the constructs.

## Conclusion

In this paper we showed that waste materials such as keratin hydrolizates derived from chicken feathers can be reused to develop a novel biomaterial, whose chemical, physical and mechanical properties can be tuned varying the gelatin content. Moreover, these materials can be processed by electrospinning system and the mechanical and biological tests showed that they could have promising applications in the tissue engineering, regenerative medicine, and biofabrication areas.

## Data Availability

The datasets generated for this study are available on request to the corresponding author.

## Author Contributions

GF, FD, and CD: writing—original draft preparation. GF, FD, SB, AD, FB, AL, DB, and PB: investigation. CM, GF, CD, and FM: data analysis. PB and GV: conceptualization and funding acquisition.

### Conflict of Interest Statement

The authors declare that the research was conducted in the absence of any commercial or financial relationships that could be construed as a potential conflict of interest.
